# Supplementary material for: Causal Relationship between Aging and Anorexia Nervosa: A White-Matter-Microstructure-Mediated Mendelian Randomization Analysis
Source: Biomedicines. 2024 Aug 16;12(8):1874. doi: 10.3390/biomedicines12081874 (PMC11351342; doi:10.3390/biomedicines12081874)
Supplement: Supplementary file 1 [file biomedicines-12-01874-s001.zip › Supplementary Figure/Figure S4/Singe SNP plot of MR analysis for TL to ubm-b-1603.pdf]

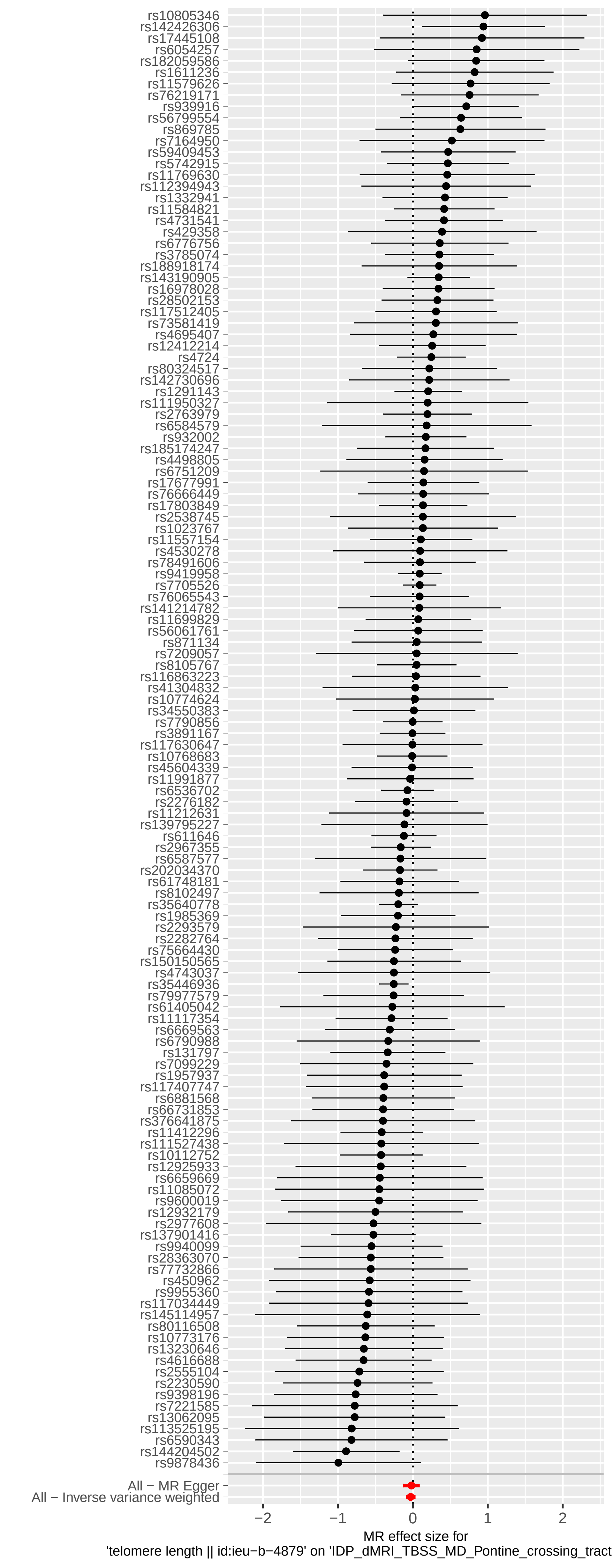

rs10805346
rs142426306
rs17445108
rs6054257
rs182059586
rs1611236
rs11579626
rs76219171
rs939916
rs56799554
rs869785
rs7164950
rs59409453
rs5742915
rs11769630
rs112394943
rs1332941
rs11584821
rs4731541
rs429358
rs6776756
rs3785074
rs188918174
rs143190905
rs16978028
rs28502153
rs117512405
rs73581419
rs4695407
rs12412214
rs4724
rs80324517
rs142730696
rs1291143
rs111950327
rs2763979
rs6584579
rs932002
rs185174247
rs4498805
rs6751209
rs17677991
rs76666449
rs17803849
rs2538745
rs1023767
rs11557154
rs4530278
rs78491606
rs9419958
rs7705526
rs76065543
rs141214782
rs11699829
rs56061761
rs871134
rs7209057
rs8105767
rs116863223
rs41304832
rs10774624
rs34550383
rs7790856
rs3891167
rs117630647
rs10768683
rs45604339
rs11991877
rs6536702
rs2276182
rs11212631
rs139795227
rs611646
rs2967355
rs6587577
rs202034370
rs61748181
rs8102497
rs35640778
rs1985369
rs2293579
rs2282764
rs75664430
rs150150565
rs4743037
rs35446936
rs79977579
rs61405042
rs11117354
rs6669563
rs6790988
rs131797
rs7099229
rs1957937
rs117407747
rs6881568
rs66731853
rs376641875
rs11412296
rs111527438
rs10112752
rs12925933
rs6659669
rs11085072
rs9600019
rs12932179
rs2977608
rs137901416
rs9940099
rs28363070
rs77732866
rs450962
rs9955360
rs117034449
rs145114957
rs80116508
rs10773176
rs13230646
rs4616688
rs2555104
rs2230590
rs9398196
rs7221585
rs13062095
rs113525195
rs6590343
rs144204502
rs9878436
All − MR Egger
All − Inverse variance weighted
−2
−1
0
1
2
MR effect size for
'telomere length || id:ieu−b−4879' on 'IDP_dMRI_TBSS_MD_Pontine_crossing_tract
